# Using the Analytic Network Process Method for Prioritizing and Weighing Shift Work Disorders Among the Personnel of Hospitals of Kerman University of Medical Sciences

**DOI:** 10.5334/jcr.163

**Published:** 2018-10-04

**Authors:** Sajad Zare, Hossein Elahi Shirvan, Rasoul Hemmatjo, Mohammad Faridan, Masoud Hajghani, Behzad Fouladi Dehaghi

**Affiliations:** 1 Department of Occupational Health, School of Public Health, Kerman University of Medical Sciences, Kerman, IR; 2 Student Research Committee, Kerman University of Medical Sciences, Kerman, IR; 3 Department of Occupational Health, School of Public Health, Urmia University of Medical Sciences, Urmia, IR; 4 Department of Occupational Health, School of Public Health, Lorestan University of Medical Sciences, Lorestan, IR; 5 Master of Science Industrial Engineering, Department of Industrial Engineering, Shahid Bahonar University of Kerman, Kerman, IR; 6 Department of Occupational Health, School of Public Health, Ahvaz Jundishapur University of Medical Sciences, Ahvaz, IR

**Keywords:** Shift work, Sleep disorders, Digestive disorders, ANP, Hospital

## Abstract

**Introduction:**

Increasing population, the need for services, and industrialization of societies have led to a growing demand for shift work. Shiftwork causes several disorders, and determining the weight of each disorders is important for their prevention and treatment. Therefore, the purpose of the present study was to use Analytic Network Process (ANP) to prioritize and weigh shift work disorders among the personnel of hospitals of Kerman University of Medical Sciences.

**Methods:**

This cross-sectional, descriptive-analytical study was conducted in 2017 among 300 shift work personnel of 10 public hospitals affiliated with Kerman University of Medical Sciences. ANP was used to prioritize and weigh shift work disorders. To this end, the criteria, sub-criteria, and alternatives were initially identified. Then, shift work disorders were categorized into 7 general criteria, 20 sub-criteria, and 3 alternatives. After designing the ANP and determining the effect of each criterion on the sub-criteria, the ANP questionnaire was developed and administered among the shift work personnel, who filled it out based on ANP. Super Decisions was subsequently used to weigh and prioritize shift work disorders.

**Results:**

The results indicated that shift work disorders among the nurses included sleep disorders (0.297), psychological disorders (0.275), digestive disorders (0.137), personal life disorders (0.122), etc., in that order of weighing. With respect to the support staff, the major shift work disorders involved sleep disorders (0.252), digestive disorders (0.198), personal life disorders (0.168), and psychological disorders (0.164). Considering security personnel, the top four shift work disorders were sleep disorders (0.201), digestive disorders (0.186), psychological disorders (0.174), and personal life disorders (0.145).

**Conclusion:**

According to the findings, sleep disorders had the highest weight in the three studied groups. Moreover, the night shift had the most profound effect on shift work disorders among the personnel in the three groups. It was followed by the evening shift. Morning shift had the lowest influence on shift work disorders. Therefore, the schedules should be taken to prevent these complications in the shift workers. It is suggested that work shift complications be included in the periodic examination program and, in case of discovery of any rhythmic disorder in each shift workers, the person should not remain in the shiftwork group for some time.

## Introduction

One of the inevitable consequences of technological advancements is shift work, which has exerted a lot of harmful influence in various occupations such as the petrochemical industry, steel industry, power plants, and essential services like police and hospital crew [1]. Generally, shift work refers to any kind of job duties that are performed outside a regular 8 to 4 job. It includes nonstandard and unnecessary responsibilities such as auditing the site and cargo transportation, half of which are fulfilled in irregular times periods [2,3,4].

Almost one fifth of workers across the globe are involved in night shift work in various industries including protective services, transportation, health care, and food preparation [5]. According to the International Labor Organization (ILO), around 15% to 20% of the workforce in developing countries and nearly 25% of the workers in developed countries are engaged in shift work [6]. Released statistics indicate that over 2.5% of American workers are involved in work that includes rotating shifts [7]. It has been reported that about 20% of the workforce in Europe is dealing with shift work too [8]. Furthermore, around 16% of workers in Australia are employed in occupations that include night shift work [9].

Shift Work Disorder (SWD), which negatively affects circadian rhythms, entails a long-term mismatch between shift workers’ sleep-wake schedule and their circadian clock. Such a mismatch may clinically lead to insomnia and/or Excessive Sleepiness (ES) [10]. Shift work can also have both psychological-social and physiological consequences [11]. With regard to physiological functions, shift work may cause a decline in working efficiency, health deterioration (e.g., disrupted sleeping and eating schedules), and disturbance of gastrointestinal, neuropsychological and cardiovascular functions [12]. As for psychological-social functions, fatigue and anxiety are also believed to stem from shift work [13].

Esquirol and colleagues demonstrated that shift work causes anxiety as well as metabolic and digestive disorders [14]. Golubic and colleagues also indicated that being involved in shift work not only leads to anxiety, but also causes various disorders such as low efficiency and stress and also job dissatisfaction [15]. Further, Chung and colleagues showed that long-term involvement in shift work leads to disorders in the sleep pattern and sympathetic system [16]. Also Choobineh and colleagues concluded that shiftwork causes disorders of musculoskeletal and digestive systems [4]. In another study, they assessed the impact of shift schedule change in a petrochemical company and mentioned the prevalence of mental problems, knee and neck sore among shift workers [17,18]. Bolghanabadi and colleagues mentioned the relation between shiftwork and increased occupational accidents among sugar factory workers [19].

Nurses are highly likely to suffer from psychological and physiological problems such as depression, anxiety, and fatigue. Nurses are involved in irregular shift works during three shifts (morning, evening and night), and because they have more direct contact with patients they are exposed to extensive psychological tension in comparison with people in other occupations. The American Association of Occupational Health Nurses has categorized nursing among the top four occupations with high prevalence of tension-related diseases and the number one health care-related job in terms of frequent exposure to tension [20,21,22].

Analytic network process (ANP) is a mathematical theory, developed by Thomas L. Saaty, to identify decision-making priorities of multiple variables without establishing one-way hierarchical relationship among decision levels [15], which has been successfully applied in various areas [14]. ANP is an attempt to improve analytic hierarchy process (AHP) based on the analysis conducted by human brain for complicated issues with non-hierarchical structures [16]. Using a comprehensive framework, ANP takes into account all the interactions and relationships among decision-making levels, which form a network structure [23].

To date, numerous studies have addressed problems caused by shift work in refineries, petrochemical complexes, health centers, and other work environments across the world. However, few research projects have tried to examine shift work disorders among hospital personnel using ANP. This method divides work shift disorders to several different levels, and disorders of each level are weighted separately to help choose the best shift.

Given that nurses provide health care services all day long, their physical and mental health is of utmost importance. Since they work in irregular shifts, their physiological, psychological, personal, and social life may be negatively influenced, a hazard, which may have consequences on nurses’ performance and efficiency [24]. These consequences may directly affect patients who are the major recipients of services provided by nurses [15]. If measures are taken to enhance nurses’ health, they would be able to provide better quality services to patients. This study sought to weigh and prioritize the major work shift disorders among personnel of the hospitals affiliated with Kerman University of Medical Sciences in Iran.

## Material and Methods

### Study design

This cross-sectional, descriptive-analytical study was conducted in 2017 among the shift work personnel of 10 public hospitals affiliated with Kerman University of Medical Sciences. The participants had at least one year of work experience. Moreover, those staff members that had a history of sleep disorder, asthma, diabetes, coronary artery disease, mental disorders, epilepsy, gastrointestinal disorders, alcohol abuse, or drug addiction were excluded from the study because the above-mentioned medical problems influence adaptation to shift work. The selected participants were divided into three groups in the light of their shift work pattern: the first group included nurses who followed 8-hour shifts with a 3-3-3-3 (3 mornings, 3 evenings, 3 nights, and 3 days off) pattern; the second group entailed support staff who worked in 12–24 hour shifts (12 hours of work followed by 24 hours of resting); and the third group encompassed security personnel who were engaged in 24–48 hour shift patterns (24 hours of work followed by 48 hours of resting).

### Sampling procedure

Two-step cluster sampling technique was used to select the participants. Based on the correlation coefficient formula with a significance level of 0.95 and a test power of 0.90, the sample size was found to be 300. Thus, 300 personnel of the hospitals were randomly selected and categorized in the three groups (100 nurses, 100 support staff members, and 100 security staff members). All the participants then completed the designed ANP questionnaire.

### ANP method

Saaty (1996) presented ANP as a generalized form of AHP [25]. In AHP, hierarchical structure and relationship among factors are required. AHP does not provide the possibility of investigating the interdependent relationships within a cluster of factors. In contrast, ANP allows the examination of interrelationships among elements, hence moving beyond linear relations [26]. It is a network-based system that replaces one-way relationships with dependencies and feedback [25,27,28]. As a result, ANP is stronger than AHP in making decisions in uncertain and dynamic environments [29].

### The following steps are followed in ANP

#### Selection of criteria, sub-criteria, and alternatives

In order to prioritize and weigh shift work disorders by the use of ANP, first, the criteria, sub-criteria, and alternatives should be determined. To this end, shift work disorders were classified into 7 criteria, 20 sub-criteria, and 3 alternatives following literature review and the standard survey of shift workers (SOS) (Table 1). SOS, which was developed by Medical Research Council/Economic and Social Research Council and Applied Psychology Unit in the United Kingdom, is one of the most valid and thorough surveys for studying shift work problems [30].

**Table 1:** Criteria and sub-criteria of ANP.

Criteria	Sub-criteria

Sleep disorders	Sleeplessness or frequent wake-up during sleep Use of hypnotics
Personal life disorders	Rest Exercise
Family life disorders	Lack of enough time for doing household chores Attending to children and parents Attendance in familial ceremonies
Mental disorders	Headache and dizziness Anger Impatience Depression
Digestive disorders	Increased/decreased appetite Constipation Ulcers
Cardiovascular disorders	Dyspnea High blood pressure Low blood pressure
Musculoskeletal disorders	Sore neck Backache Sore leg and knee

The study also included three alternatives: morning shift, evening shift, and night shift. Because workers experienced these three shifts in the past, these three alternatives (shifts) are chosen for assessing the healthcare in different shifts. The shift workers with the lowest scores would be the ones with fewer disorders.

#### The procedure for network design

**Figure 1:**
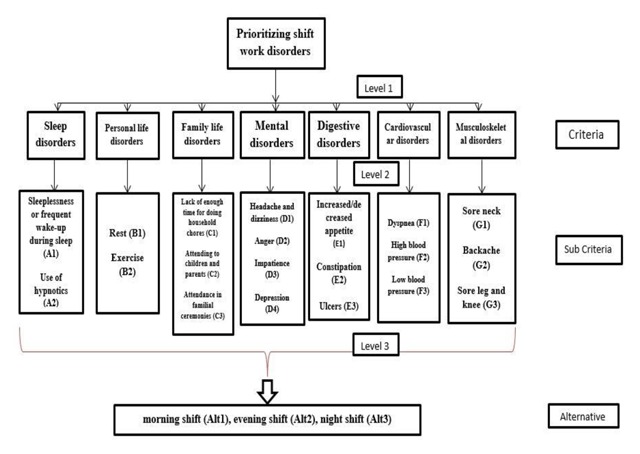
The network structure for prioritizing and weighing.

At first, the researchers developed a matrix in which the lines and columns included factors that might cause shift work disorders. Then, it was presented to domain experts who were requested to identify interrelationships among influential factors (Figure 1).

#### Establishment of pairwise comparison matrices

Upon constructing the model, a pairwise comparison matrix was developed using the appropriate Saaty scale given in Table 2, *C* = 〈*C_j_|j* = 1, 2, …, *n*〉. This pairwise comparison of “n” criteria *a_ij_*(*i, j* = 1, 2, …, *n*) yields an (n × n) evaluation matrix ***A***, in which each element is the quotient of criteria weights, as illustrated:

1 A = \left[{\begin{array}{*{20}{c}}
{{a_{11}}}&{{a_{12}}}& \cdots &{{a_{1n}}}\\
{{a_{21}}}&{{a_{22}}}& \cdots &{{a_{2n}}}\\
\vdots & \vdots & \ddots & \vdots \\
{{a_{n1}}}&{{a_{n2}}}& \cdots &{{a_{nn}}}
\end{array}} \right]

2 {a_{ii}} = 1,\;{a_{ij}} = 1/{a_{ij}},\;{a_{ij}} \ne 0

At the end, a mathematical process began to normalize and detect relative weights of each matrix, which are given by the right Eigen vector (w) associated with the largest Eigen value (λ):

3 {A_w} = {\lambda _{\max}}w

**Table 2:** Pairwise comparison scale.

Scale of importance	Crisp score	Reciprocal of crisp score

Equal importance	1	1
Moderate	3	.33
Strong importance	5	.20
Very strong importance	7	.14
Extremely preferred	9	.11

The quality of ANP output largely depends on the consistency of the pairwise comparison of judgments. The consistency has to do with the relation between the entries of *A*: *a_ij_* × *a_jk_* = *a_ik_*. The Consistency Index (CI) is obtained through the following formula:

4 CI = ({\lambda _{\max}} - n)/(n - 1)

The pairwise comparison was normalized and priority vector was computed to weigh the matrix elements. The values of these vectors were summed to 1. Consistency ratio (CR) can be calculated to estimate the consistency of the subjective input in the pairwise comparison matrix. Generally, if CR < 0.1, it is an acceptable index [31]. The CR for each square matrix was obtained through dividing the CI values by Random Consistency Index (RCI) values.

5 CR = {{CI}/{RI}}

The RCI is obtained from a larger number of simulations and varies in the light of the matrix order [31]. Table 3 illustrates the RCI values for matrices of the order varying from 1 to 10. They were obtained by approximating random indices using a sample size of 500. Acceptable CR ranges depend on the matrix size. If CR is bigger than an acceptable value, there will be inconsistent judgments within that matrix, meaning that the evaluation process should be reviewed, reconsidered, and improved.

**Table 3:** Average RCI based on matrix size.


**S. no**	1	2	3	4	5	6	7	8	9	10
**RCI**	0	0	.52	.89	1.11	1.25	1.35	1.40	1.45	1.49


#### Pairwise comparison matrices of interdependencies

A pairwise comparison was constructed among all attributes-enablers, and corresponding values were calculated to compare all the interdependencies in a network.

#### Formation and analyzing of super matrix

The super matrix is utilized to represent the various interdependent effects existing between different process elements. It shows the association and interdependencies between criteria and sub-criteria.

In the super matrix, which is a partitioned matrix, the sub-matrix consists of relationships between graphical models. Since its Eigen vectors may not be equal to 1, the super matrix will be regarded as an unweighted matrix. The super matrix should be converted into reasonable priorities. To do so, the unweighted matrix was multiplied by the priority weights generated from the clusters, yielding the weighted matrix. The weighted super matrix was subsequently raised to a considerably large power to have converged or stable values. This was achieved by raising the super matrix to the power 2K+1, where k is an arbitrary large number or it can be discovered using the ANP solver. The obtained matrix is known as the limit matrix.

#### Computation of desirability index

The following equation was used to calculate the desirability index:

6 {D_i} = \sum\limits_{j = 1}^j {\sum\limits_{k = 1}^{kj} {{P_j}A_{kj}^DA_{kj}^1{S_{ikj}}}}

Where *P_j_* is the relative importance weight of criteria *j*; A_{kj}^D is the relative weight for sub-criteria *k* of *j* for the dependency; A_{kj}^1 is the stabilized relative importance weight for sub criteria *k* of criteria *j* for the independency; *S*_1*kj*_ is the relative impact of strategy alternative 1 on sub-criteria *k* of criteria *j* of maintenance strategy selection network; *S*_2*kj*_ is the relative impact of strategy alternative 2 on sub-criteria *k* of criteria *j* of maintenance strategy selection network; *S*_3*kj*_ is the relative impact of strategy alternative 3 on sub-criteria *k* of criteria *j* of maintenance strategy selection network; and *S*_4*kj*_ is the relative impact of strategy alternative 4 on sub-criteria *k* of criteria *j* of maintenance strategy selection network.

#### Designing the questionnaire and an instrument to weigh and prioritize shift work disorders

After designing the ANP network and determining the effect of each criterion on the sub-criteria, the ANP questionnaire was developed and administered among the shift work personnel, who filled it out based on ANP. Super decisions was subsequently used to weigh and prioritize shift work disorders.

#### Ethical considerations

The researchers obtained ethical approval from the Ethics Committee of Kerman University of Medical Sciences (ID: IR.KMU.REC.1396.1783). All participating workers were required to sign a consent form prior to taking part in the study, and also agreeing to participate voluntary in this study.

## Results

### Demographic information

Table 4 displays the demographic information of the study sample.

**Table 4:** Demographic information of the study sample (n = 300).

Variables		Frequency	Percentage

Gender	Male	300	100

Marital status	Single	84	28
	Married	216	72

Work experience	Less than 5 years	95	31.6
	5–10 years	103	34.4
	11–20 years	67	22.4
	21–30 years	35	11.6

Age range	24–30 years	130	43.4
	31–40	96	32
	41–50	74	24.6

Shift work selection	Voluntary	54	18
	Obligatory	246	82

Shift work satisfaction	Yes	186	62
	No	114	38

### Normalized weight of shift work disorders (criteria), sub-criteria, and alternatives in the three groups

Table 5 represents the normalized weight of shift work disorders (criteria) and sub-criteria for nurses working 8-hour shifts. It is observed that the shift work disorders are mainly manifested in the form of sleep disorders (0.297), mental disorders (0.275), digestive disorders (0.137), and personal disorders (0.122).

**Table 5:** Normalized weight for shift work disorders (criteria) and sub-criteria in the first group (nurses working 8-hour shifts).

Group	Criterion	Criterion weight	Sub-criterion	Sub-criterion weight

The first group: nurses working 8-hour shifts	Sleep disorders	0.297	Sleeplessness or frequent wake-up during sleep	0.633
			Use of hypnotics	0.366
	Personal life disorders	0.122	Rest	0.634
			exercise	0.365
	Family life disorder	0.113	Lack of enough time for doing household chores	0.745
			Attending to children and parents	0.146
			Attendance in familial ceremonies	0.108
	Mental disorders	0.275	Headache and dizziness	0.052
			Anger	0.194
			Impatience	0.133
			Depression	0.619
	Digestive disorders	0.137	Increased/decreased appetite	0.273
			Constipation	0.104
			Ulcers	0.621
	Cardiovascular diseases	0.032	Dyspnea	0.600
			High blood pressure	0.082
			Low blood pressure	0.317
	Musculoskeletal disorders	0.024	Sore neck	0.089
			Backache	0.632
			Sore leg and knee	0.278

Sleep disorders are majorly materialized in the form of sleeplessness or frequent wake-up during sleep (0.633) followed by the use of hypnotics (0.366). Considering mental disorders, the largest weight was recorded for depression (0.619), followed by anger (0.194), impatience (0.133), and headache and dizziness (0.052).

The final results of ranking alternatives (the type of shift work) based on shift work disorders among nurses are presented below:

Night shift work (0.656) has the most profound effect on the incidence of disorders, followed by evening shift work (0.183) and morning shift work (0.160) in that order.

Normalized weight of shift work disorders (criteria), sub-criteria, and alternatives in the second group: support personnel working 12–24 hour shift patterns.

The normalized weight of shift work disorders (criteria) and sub-criteria for the support personnel working 12–24 hour shift patterns are presented in Table 6. As observed, the effect of shift work is majorly detected in sleep disorders (0.252), digestive disorders (0.198), personal life disorders (0.167), and mental disorders (0.164) in that order.

**Table 6:** Normalized weight for shift work disorders (criteria) and sub-criteria in the second group (support personnel working 12–24 hour shift patterns).

Group	Criterion	Criterion weight	Sub-criterion	Sub-criterion weight

The second group: support personnel working 12–24 hour shift patterns	Sleep disorders	0.252	Sleeplessness or frequent wake-up during sleep	0.546
			Use of hypnotics	0.454
	Personal life disorders	0.167	Rest	0.567
			exercise	0.433
	Family life disorder	0.120	Lack of enough time for doing household chores	0.672
			Attending to children and parents	0.116
			Attendance in familial ceremonies	0.212
	Mental disorders	0.164	Headache and dizziness	0.095
			Anger	0.236
			Impatience	0.147
			Depression	0.522
	Digestive disorders	0.198	Increased/decreased appetite	0.284
			Constipation	0.215
			Ulcers	0.501
	Cardiovascular diseases	0.067	Dyspnea	0.526
			High blood pressure	0.196
			Low blood pressure	0.278
	Musculoskeletal disorders	0.033	Sore neck	0.329
			Backache	0.475
			Sore leg and knee	0.196

The final results of ranking alternatives (the type of shift work) based on shift work disorders among support personnel are presented below:

Night shift work (0.672) has the most profound effect on the incidence of disorders, followed by morning shift work (0.328).

Normalized weight of shift work disorders (criteria), sub-criteria, and alternatives in the third group: security personnel working 24–48 hour shift patterns.

Table 7 shows the normalized weight of shift work disorders (criteria) and sub-criteria for the security personnel working 24–48 hour shift patterns.

**Table 7:** Normalized weight for shift work disorders (criteria) and sub-criteria in the third group (security personnel working 24–48 hour shift patterns).

Group	Criterion	Criterion weight	Sub-criterion	Sub-criterion weight

The second group: support personnel working 12–24 hour shift patterns	Sleep disorders	0.201	Sleeplessness or frequent wake-up during sleep	0.785
			Use of hypnotics	0.215
	Personal life disorders	0.145	Rest	0.477
			exercise	0.523
	Family life disorder	0.121	Lack of enough time for doing household chores	0.411
			Attending to children and parents	0.361
			Attendance in familial ceremonies	0.228
	Mental disorders	0.174	Headache and dizziness	0.109
			Anger	0.278
			Impatience	0.198
			Depression	0.415
	Digestive disorders	0.186	Increased/decreased appetite	0.312
			Constipation	0.206
			Ulcers	0.482
	Cardiovascular diseases	0.075	Dyspnea	0.389
			High blood pressure	0.227
			Low blood pressure	0.384
	Musculoskeletal disorders	0.098	Sore neck	0.175
			Backache	0.423
			Sore leg and knee	0.402

It is observed that the negative impact of shift work was profoundly detected in sleep disorders (0.201), digestive disorders (0.186), mental disorders (0.174), and personal life disorders (0.145).

The final results of ranking alternatives (the type of shift work) based on shift work disorders among security personnel are presented below:

Night shift work (0.672) has the most profound effect on the incidence of disorders, followed by evening shift work (0.323) and morning shift work (0.265).

## Discussion

This study aimed at prioritizing and weighing shift work disorders among shift workers of hospitals affiliated with Kerman University of Medical Sciences in 2017 using ANP. To date, many studies have investigated the effect of shift work on workers in refineries, petrochemical complexes, health centers, and other work environments across the world. Nonetheless, studies on prioritizing and weighing shift work disorders using ANP are scanty.

According to the obtained results, the most important observable disorders among nurses who worked in 8-hour shifts include sleep disorders, mental disorders, digestive disorders, and personal life disorders. With respect to sleep disorders, the major type of disorder was sleeplessness or frequent wake-up during sleep, followed by the use of hypnotics. Regarding mental disorders, the major effects were detected in depression, anger, impatience, and headache/dizziness in that order (Table 5). Considering support personnel, shift work mainly led to sleep disorders, digestive disorders, personal life disorders, and mental disorders. With regard to sleep disorders, the most important type of disorder was sleeplessness or frequent wake-up during sleep. Moreover, considering digestive disorders, the major types of disorders involved ulcers, increased/decreased appetite, and constipation (Table 6). Focusing on security personnel, shift work disorders were mainly materialized in sleep disorders, digestive disorders, mental disorders, and personal life disorders. With respect to sleep disorders, the two main types of disorders were sleeplessness or frequent wake-up during sleep and use of hypnotics. Further, the major types of digestive disorders included ulcers, increased/decreased appetite, and constipation, in that order (Table 7). Thus, the main type of disorder observed among all studied shift workers was sleep disorder. The normalized weights of this disorder type among nurses, support personnel, and security personnel were 0.297, 0.252, and 0.201, respectively.

It is observed that nurses suffered more from sleep disorders in comparison with the other two groups. The second type of disorder caused by shift work among nurses was mental disorder with a normalized weight of 0.275. On the other hand, the second major shift work disorder among support and security personnel was digestive disorder with normalized weights of 0.198 and 0.186 respectively. The third important disorder type among nurses was digestive disorder (with a weight of 0.137), while that of support personnel was personal life disorder (0.167). Additionally, mental disorder was the third major disorder type among security personnel (0.174).

Studying shift work disorders among operating room technicians of hospitals affiliated with Shiraz University of Medical Sciences in 2007, Choobineh et al. concluded that mental disorders, digestive disorders, and social life disorders had the highest prevalence among the participants with proportions of 97.6%, 70.6%, and 66.5%, respectively. They attributed these disorders to the mismatch between shift workers’ work schedule and circadian rhythm [32]. The results of their study are slightly different from the ones obtained in the current research. More precisely, we found that mental disorders constituted the second major type of disorder among nurses; in addition, the second main type of disorder among support and security personnel involved digestive disorders.

Zamanian and colleagues [33] examined shift work disorders among security personnel of three hospitals affiliated with Shiraz University of Medical Sciences. Comparing systolic (p = 0.011) and diastolic (p = 0.012) blood pressure among the control group (security personnel of the morning shift and office workers) and the case group (shift workers) showed that the latter had higher blood pressure than the former. They also indicated a significant difference in the sleep quality of the two groups, meaning that the case group suffered from lack of enough sleep (p = 0.018) and sleeplessness (p = 0.001). Comparing the effect of shift work on personal and family life of the two groups (p = 0.001), the researchers argued that shift work had a more profound impact on both personal and family life of shift workers. They also discovered a measurable difference between the two groups in terms of digestive disorders (p = 0.015), demonstrating that shift workers suffered from increased appetite (p = 0.015) and ulcers (p = 0.035). Comparison of musculoskeletal disorders, social life disorders, mental disorders, and cardiovascular disorders between the two groups also displayed significant differences (p = 0.28, p = 0.001, p = 0.001, and p = 0.001, respectively). In all these types of disorders, shift workers were in a much worse condition [33]. The findings of this study are very much in line with our results, indicating that digestive disorders, mental disorders, and personal/social life disorders are the consequences of shift work. Garbarino [34] investigated the influence of shift work on the health and safety in the work environment and showed that shift work causes disorders in social and family life and that it negatively affects people’s performance and social relations. The negative impacts of shift work generally are chronic, hence increasing the risk of neurological, cardiovascular, intestinal/stomach diseases [34]. The results of the present study further confirm this claim. De Bacquer and colleagues [23] studied the association between rotating shift work and the metabolic syndrome, concluding that shift work enhances occupational tension and the risk of digestive disorders. The findings of this study are very much in line with our results and digestive disorders are high in these three groups. Other research teams showed that disorders in biological rhythm of the body caused by shift work and night shift work result in different diseases like digestive and musculoskeletal disorders [35,36]. These results are in line with ours, in that, we also found that disorders were particularly prevalent among night shift workers.

Asghari and colleagues [3] examined work shift disorders among automobile manufacturing workers and concluded that 82% of the participants had digestive disorders, with the most frequently mentioned problem being reduced appetite, stomachache, and heartburn. Moreover, 30.7% of the respondents said that they always suffered from lack of sleep. In addition, 43.3% and 26% respectively said that they often suffered from sleeplessness. On the other hand, 78% were not happy with the proportion of time they spent with their family and 86.7% believed that shift work had negatively affected their personal life. Additionally, 87.3% reported that shift work had affected their family life, while 86% believed that it had negatively influenced their social life. In addition, 58% attributed their distraction to shift work. Moreover, 51.5% of the participants believed that they suffered from chronic fatigue during the day, whereas 38.7% reported that they were aggressive. Finally, 10.7% said that they suffered from cardiovascular diseases [3]. The results of this study are similar to our findings.

## Conclusion

Based on the findings, working during the night shift has the most negative effect on shift work disorders. It is followed by evening and morning shifts in that order. As a result, the best work shifts in terms of lower disorder incidence are the morning shift and evening shift. Night shift is the worst work shift in this regard. Therefore, it is suggested that managers should make decisions to reduce these disorders as much as possible. In addition, it is advised to use other multi-criteria decision-making methods to solve this problem, such as VIKOR and PROMETHEE.
